# An Unusual Complication of Electronic Cigarette Use: Missed Inhaled Foreign Body Causing Acute Respiratory Failure

**DOI:** 10.7759/cureus.15731

**Published:** 2021-06-18

**Authors:** Saarth Shiralkar, James Fletcher, Madhu Balasubramaniam

**Affiliations:** 1 Department of Anaesthetics and Critical Care, Royal Bolton Hospital, Bolton, GBR; 2 Department of Cardiothoracic Anaesthesia, Critical Care and ECMO, Wythenshawe Hospital, Manchester, GBR

**Keywords:** case report, electronic cigarette, extracorporeal membrane oxygenation, foreign body aspiration, persistent pneumonia

## Abstract

We present a case of a young woman who was admitted to the hospital with persistent pneumonia and cough productive of purulent green sputum. She was admitted to the intensive care unit due to type 1 respiratory failure. Chest computerised tomography (CT) showed a large right-sided hydropneumothorax, for which a right-sided chest drain was inserted. Despite intubation, oxygenation continued to deteriorate and the patient was commenced on veno-venous extracorporeal membrane oxygenation (ECMO) and transferred to the regional ECMO centre. Bronchoscopy revealed a plastic coil from an electronic cigarette at the entrance to the right lower lobe. Following its removal, the patient’s condition rapidly improved and she was successfully weaned from ECMO and discharged from the cardiothoracic critical care unit. There are very few reports of tracheobronchial foreign body (FB) aspiration secondary to electronic cigarette use, and tracheobronchial FB aspiration in adults requiring veno-venous ECMO to treat respiratory failure is uncommon. This case highlights the importance of considering tracheobronchial FB aspiration as a potential diagnosis in patients who present with more than two weeks of pneumonia not responding to treatment.

## Introduction

Tracheobronchial aspiration of foreign bodies (FBs) is common in children, especially those under 3 years old [1]. However, instances of tracheobronchial FB aspiration are uncommon in adults and tend to occur in older adults or those with underlying health conditions such as significant neurological impairment [2,3]. Organic FBs, such as bone fragments and seeds, are more frequently aspirated than inorganic FBs, such as small plastic objects [1]. Complications of tracheobronchial aspiration of FBs include pneumomediastinum, hydropneumothorax, lung abscess, pneumonia, bronchial stenosis, and pneumothorax [4]. These complications occur due to lung mucosal inflammation, granulation tissue formation, and tissue injury [5]. Here, we present a case of an unknown tracheobronchial aspiration of a small plastic coil from an electronic cigarette, resulting in hydropneumothorax and severe acute respiratory failure requiring extracorporeal membrane oxygenation (ECMO).

## Case presentation

A 22-year-old female with well-controlled asthma and a smoking history presented to her General Practitioner with increasing shortness of breath and cough; she was treated as presumed pneumonia with antibiotics and steroids. She presented to Royal Bolton Hospital (RBH) Emergency Department (ED) four days later with ongoing symptoms. Examination findings included right-sided chest pain and right basal crepitations. The patient declined hospital admission and was discharged with clarithromycin. Two weeks later, she presented for a second time to RBH ED with shortness of breath and cough productive of green, offensive smelling sputum. Chest radiograph demonstrated significant right-sided consolidation; she was admitted under the Acute Medical team and treated as community-acquired pneumonia and possible empyema. Her current antibiotics were continued and she was due to be revived by the Respiratory team the following day for consideration for thoracocentesis.

**Figure 1:**
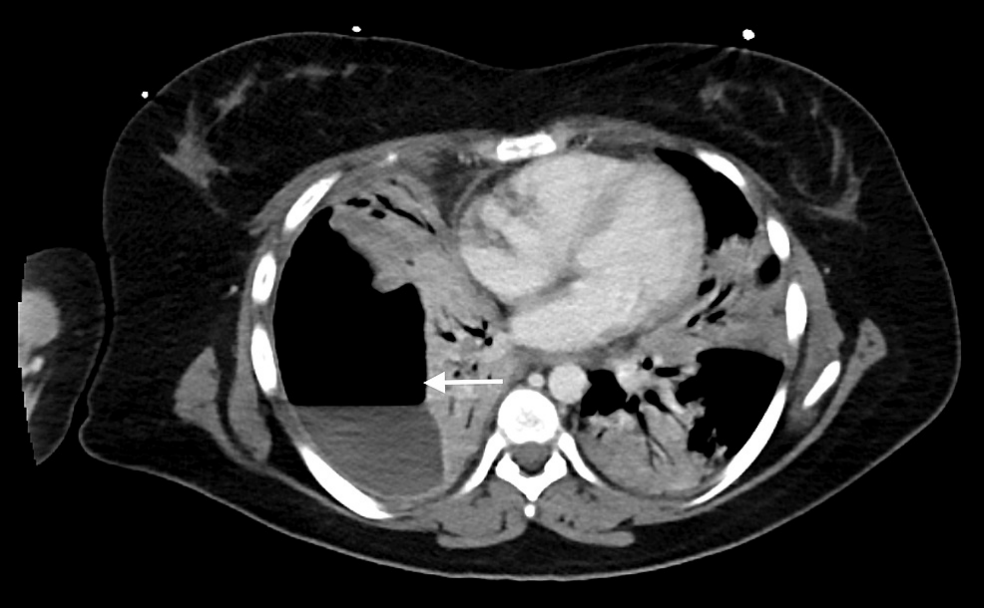
Chest CT prior to insertion of right-sided chest drain showing large hydropneumothorax (arrow)

Within 12 hours of admission, the patient became profoundly hypoxic and she was admitted to the intensive care unit (ICU). She complained of worsening right-sided chest pain, dyspnoea, pyrexia, and copious purulent secretions. Examination revealed bibasal crackles and reduced air entry to the right lung. Oxygen saturations improved with expectoration. Laboratory tests showed elevated inflammatory markers and urine culture was positive for pneumococcal antigen; following discussion with Microbiology, the patient was commenced on piperacillin-tazobactam and clarithromycin. A SARS-CoV-2 polymerase chain reaction of nasopharyngeal swabs was negative. The patient was commenced on 100% high-flow oxygen therapy at 20 litres to treat her type 1 respiratory failure, which became type 2 respiratory failure as she began to tire. Chest computerised tomography (CT) demonstrated a large right-sided hydropneumothorax with associated right upper and lower lobe collapse, and bilateral florid consolidation (Figure 1). Following discussion with the Respiratory team, a right-sided chest drain was inserted, which initially drained air followed by cloudy liquid.

Despite the chest drain, the patient continued to deteriorate with her high-flow oxygen requirement increasing to 80 litres, and a decision was made to intubate. Shortly after intubation, the patient suffered a cardiac arrest with pulseless electrical activity. A left-sided tension pneumothorax was identified and managed with needle decompression and the patient’s condition rapidly improved; a left-sided chest drain was subsequently sited. Due to ongoing ventilatory difficulties, the patient was referred to the regional ECMO centre. The ECMO retrieval team attended and commenced her on veno-venous ECMO with cannulation of the right internal jugular and right femoral veins prior to transfer to the cardiothoracic critical care unit (CTCCU) at Wythenshawe Hospital.

On admission to the ECMO centre, antimicrobials were escalated to meropenem and clarithromycin, and acyclovir was added. She was also started on continuous veno-venous haemofiltration due to metabolic acidosis. A bronchoscopy was performed showing inflamed bronchial mucosa throughout both lungs. At the entrance to the right lower lobe, a potential FB was seen, which was found to be immovable. A referral was made to the Thoracic Surgery team in view of this finding.

**Figure 2:**
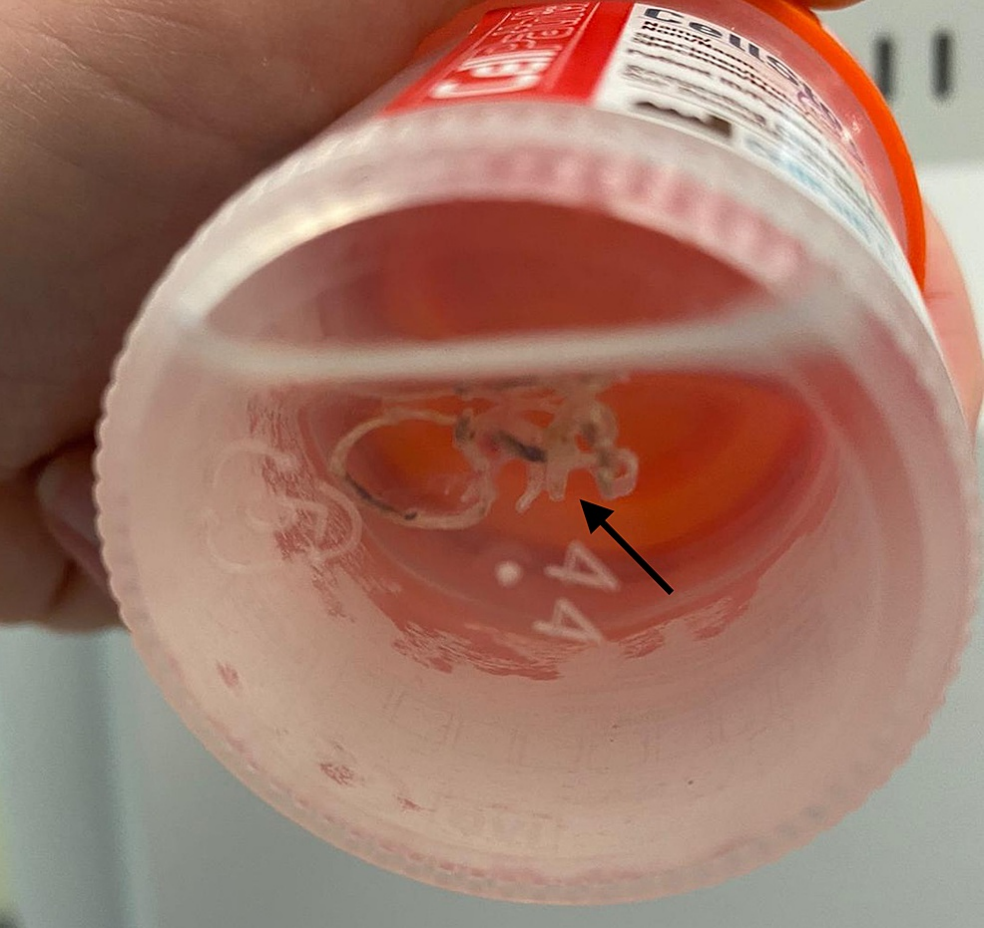
Plastic coil removed from the entrance to the right lower lobe bronchus (arrow)

The following day, rigid bronchoscopy was performed by the thoracic surgeons and the FB was successfully removed (Figure 2). On inspection, it was revealed to be a clear plastic coil from an electronic cigarette. A transthoracic echocardiogram was subsequently performed, which showed a moderately dilated and impaired right ventricle, and the patient was started on a milrinone infusion and sildenafil.

Following the removal of the FB, the patient’s condition improved significantly. Eight days after commencing on ECMO, the circuit oxygenator was removed in preparation for decannulation the following day. ECMO was weaned successfully and the patient was extubated on day 11 of admission and discharged from CTCCU two days later. On questioning, the patient denied any history of FB aspiration. She subsequently self-discharged from the hospital and did not attend her outpatient follow-up appointment.

## Discussion

This case highlights the potentially life-threatening consequences of missed tracheobronchial FB aspiration. Eliçora et al. [6] reported a case similar to ours involving aspiration of a plastic FB in a 53-year-old man with a background of smoking and COPD. As in our case, there was no evidence of the aspirated FB on the chest radiograph, and it was only identified following further investigation. Other reports exist of tracheobronchial FB aspiration in adults requiring veno-venous ECMO to treat respiratory failure [7]; however, the time between FB aspiration and respiratory failure was much more acute than in our case. The use of ECMO in the management of patients with FB aspiration is much more common in the paediatric population [8].

In this case, tracheobronchial FB aspiration was not initially considered as a possible diagnosis. The patient was 22 years old with no medical issues other than well-controlled asthma. Furthermore, she did not report any history of FB aspiration. Given the rapid improvement in the patient’s condition, once the FB was removed, we are certain that it was the cause of the patient’s persistent symptoms and ultimately respiratory failure. However, as the patient self-discharged from the hospital shortly after being stepped down to the ward from CTCCU and did not attend her outpatient follow-up appointment, it has not been possible to explore other possible causes for her respiratory failure. It has also not been possible to assess whether she has made a full recovery.

## Conclusions

Despite being rare, it is important to consider tracheobronchial FB aspiration as a potential diagnosis in patients who present with more than two weeks of pneumonia not responding to treatment. Bronchoscopy should be considered when lobar changes persist or when there is significant pus or empyema in normally fit and well individuals. Clinicians should also be aware of the potentially life-threatening consequences of missed tracheobronchial FB aspiration and that non-treatment could result in chronic disease in surviving patients.
